# Association Analysis of Noncoding Variants in Neuroligins 3 and 4X Genes with Autism Spectrum Disorder in an Italian Cohort

**DOI:** 10.3390/ijms17101765

**Published:** 2016-10-22

**Authors:** Martina Landini, Ivan Merelli, M. Elisabetta Raggi, Nadia Galluccio, Francesca Ciceri, Arianna Bonfanti, Serena Camposeo, Angelo Massagli, Laura Villa, Erika Salvi, Daniele Cusi, Massimo Molteni, Luciano Milanesi, Anna Marabotti, Alessandra Mezzelani

**Affiliations:** 1 Institute of Biomedical Technologies, National Research Council, Via Fratelli Cervi 93, 20090 Segrate, Italy; landinimartina@gmail.com (M.L.); ivan.merelli@itb.cnr.it (I.M.); nadiagalluccio@gmail.com (N.G.); daniele.cusi@unimi.it (D.C.); luciano.milanesi@itb.cnr.it (L.M.); 2 Scientific Institute, IRCSS Eugenio Medea, 23842 Bosisio Parini, Italy; mariaelisabetta.raggi@bp.lnf.it (M.E.R.); francesca.ciceri@bp.lnf.it (F.C.); arianna.bonfanti@bp.lnf.it (A.B.); laura.villa@bp.lnf.it (L.V.); massimo.molteni@bp.lnf.it (M.M.); amarabotti@unisa.it (A.M.); 3 Scientific Institute, IRCSS Eugenio Medea, 72100 Brindisi, Italy; serena.camposeo@libero.it (S.C.); angelo.massagli@gmail.com (A.M.); 4 Department of Health Sciences, University of Milan, 20142 Milan, Italy; erika.salvi@unimi.it; 5 Sanipedia srl, via Ariosto 21, 20091 Bresso, Italy; 6 Department of Chemistry and Biology, University of Salerno, Via Giovanni Paolo II 132, 84084 Fisciano, Italy

**Keywords:** autism, genetics, neuroligins, SNPs, haplotype analysis, noncoding regions

## Abstract

Since involved in synaptic transmission and located on X-chromosome, neuroligins 3 and 4X have been studied as good positional and functional candidate genes for autism spectrum disorder pathogenesis, although contradictory results have been reported. Here, we performed a case-control study to assess the association between noncoding genetic variants in *NLGN3* and *NLGN4X* genes and autism, in an Italian cohort of 202 autistic children analyzed by high-resolution melting. The results were first compared with data from 379 European healthy controls (1000 Genomes Project) and then with those from 1061 Italian controls genotyped by Illumina single nucleotide polymorphism (SNP) array 1M-duo. Statistical evaluations were performed using Plink v1.07, with the Omnibus multiple loci approach. According to both the European and the Italian control groups, a 6-marker haplotype on *NLGN4X* (rs6638575(G), rs3810688(T), rs3810687(G), rs3810686(C), rs5916269(G), rs1882260(T)) was associated with autism (odd ratio = 3.58, *p*-value = 2.58 × 10^−6^ for the European controls; odds ratio = 2.42, *p*-value = 6.33 × 10^−3^ for the Italian controls). Furthermore, several haplotype blocks at 5-, 4-, 3-, and 2-, including the first 5, 4, 3, and 2 SNPs, respectively, showed a similar association with autism. We provide evidence that noncoding polymorphisms on *NLGN4X* may be associated to autism, suggesting the key role of *NLGN4X* in autism pathophysiology and in its male prevalence.

## 1. Introduction

Autism spectrum disorder (ASD) is a complex neurodevelopmental disorder with an early onset, typically prior to age 3, characterized by impaired social interactions, absent or limited verbal communication, and stereotyped and restricted pattern of interests [1]. Considering the great variability of clinical presentations, the definition of ASD includes autism, Asperger’s syndrome, and pervasive developmental disorder not otherwise specified (PDD-NOS) [2]. A combination of both genetic and environmental causes, such as pesticides, heavy metals, dysbiosis, and mycotoxins, has been suggested for ASD pathogenesis but, despite approximately 5%–15% of ASDs cases being due to identified genetic or chromosomal alterations, the ASD etiology is still largely unknown [3,4,5,6,7,8]. Considering the higher occurrence of ASD in males than in females, with a ratio of roughly 4:1, a role for the X-chromosome in the ASD etiology has been also suggested [7,9]. In this regard, cytogenetic abnormalities highlight the putative involvement of the loci from Xq12 to Xq21 and Xp22 in autism pathogenesis [10,11], as well as associations with structural variants that have been reported for some X-chromosomal genes, such as *NLGN4X* and *MECP2* genes [12,13,14,15]. Since located on the X-chromosome and involved in synaptic plasticity, both Neuroligin3 (*NLGN3*) and Neuroligin4X (*NLGN4X*), mapped at Xq13.1 and Xp22.31, respectively, were extensively studied as good positional and functional candidates for ASD predisposing. Neuroligins are postsynaptic cell-adhesion molecules that interact with the presynaptic cell-surface receptors neurexins. Neurexins contain a single transmembrane domain, and together with neuroligins they form Ca^2+^-dependent neurexin/neuroligin complexes. These transsynaptic complexes play a crucial role in modulating neurotransmission and differentiation [16,17,18]. Several ASD genetic studies have identified mutations affecting neuroligin proteins and influencing synaptic function [19,20,21]. The involvement of neuroligins in ASD has been firstly confirmed in two Swedish families by a de novo missense mutation (R451C) in *NLGN3* and a frameshift mutation (1186insT) in *NLGN4X* causing premature protein termination (D396X), respectively associated with typical autism and Asperger’s syndrome [12]. Both these mutations resulted in an intracellular retention of the mutant proteins, with a consistent loss of the synaptic activity, leading to neurodevelopmental defects and mental retardation [22,23]. In mouse models, autism-associated *NLGN3* mutations induced repetitive behaviors, and R451C mutant mice showed impaired social interactions, but enhanced spatial learning abilities [24,25,26]. A loss-of-function mechanism has also been suggested for the point mutation R87W in *NLGN4X,* found in two brothers with classical ASD [27]. Recently, missense variations in *NLGN3* (G426S) and *NLGN4X* (G84R, Q162K, and A283T) in Chinese ASD patients have been associated to ASD predisposing, by causing abnormal synaptic homeostasis [28]. Deletion of exons 4, 5, and 6 in *NLGN4X* have been also found in autistic children, suggesting that alternative splicing variants might lead to abnormal neuroligin function in ASD [29,30]. Moreover, several noncoding genetic variants have been specifically found in ASD patients [31,32,33,34,35,36,37]. All these variations often segregate into ASD families [12,13] and can also be associated with different cognitive phenotypes, such as intellectual and language disabilities [32,37], highlighting the role of neuroligins in the ASD pathogenesis. In this regard, a de novo base pair substitution (−335G>A) in the promoter region of *NLGN4X*, has been found in one autistic child with nonsyndromic mental retardation (NSMR) [32]. Since associated with an increased level of *NLGN4X* transcripts, this variation probably affects the binding sites of transcription factors in the mutated promoter sequence [32]. Four novel synonymous substitutions, specific to ASD, have been reported in the coding sequences of the *NLGN3* (p.K566K) and *NLGN4X* (p.G99G; p.I172I; p.F530F) [34]. Despite the uncertainty of the physiological and clinical relevance, they might affect the protein structures by altering splicing sites [34] or affecting gene regulation, as they are located in conserved regulatory regions, such as enhancer- and promoter-associated histone modification sites [33]. Moreover, a positive association with ASD has been identified in a homogeneous autistic Chinese cohort (made of patients with typical phenotype), for a common intronic variant in *NLGN3* (rs4844285(G)) [6]. A three-marker haplotype (rs11795613(A), rs4844285(G), rs4844286(T)), with a significant male bias, has been suggested, supporting the hypothesis that defects of the synapse might have a role in ASD pathogenesis [6]. In addition, three haplotype sets in *NLGN4X* (rs3810686(T), rs1882260(C)), (rs6638575(G), rs3810686(T), rs18882260(T)) and (rs6638575(A), rs3810686(C), rs18882260(C)), have been positively associated with nonspecific mental retardation (Intelligence Quotients IQs < 70) and social disability scores ≤ 8 [36] and genetic variants in *NLGN4X* also have a significant effect on male cognitive abilities, highlighting the role of neuroligins in psychiatric conditions [37].

However, contradictory results have been also provided in validating the relevance and frequency of neuroligin genetic variants in ASD [38,39,40,41]. Wermenter and coworkers failed to find an involvement of *NLGN3* and *NLGN4X* with ASD in a study group of 107 probands with autistic disorders at a high-functioning level [40], and similar results have been reported in the Quebec population, after screening for neuroligin mutations in 96 individuals diagnosed with ASD [41]. Lastly, no association was found between *NLGN3/NLGN4X* alterations and ASD in a Chinese cohort [38]. However, the genetic heterogeneity of ASD, as well as differences in the ethnicity background of ASD samples, make it difficult to elucidate the involvement of neuroligin genes in autism pathogenesis.

Considering all these evidences and starting from an Italian ASD sample set, we assessed the effects of three noncoding genetic variants in *NLGN3*, already reported as associated with ASD in a Chinese cohort [6], and six common single nucleotide polymorphisms (SNPs) in *NLGN4X*, only partially described in literature [36], with the final aim to validate the selected genetic variants as susceptibility loci for ASD.

## 2. Results

### 2.1. Single-Locus Analysis

Association analyses have been performed considering SNPs located in the intronic and 3′ UTR regions of *NLGN3* and *NLGN4X*, considering both the European (EUR) population (1000 Genomes Project, Phase 2) [42] and the Italian population. Genotype counts of SNPs and results of the Hardy–Weinberg statistics, which do not present any significant disequilibrium, are shown in Tables S1 and S2. As shown in Table 1, a moderate statistical significance difference (odds ratio (OR) = 1.453, *p* = 0.0456) was identified in the single-locus analysis only for SNP rs3810688, with the minor allele T having frequency slightly lower in ASD cases in comparison to EUR controls (separate results for male and female analysis are provided in Tables S3 and S4). However, this association lost its significance after False Discovery Rate (FDR) correction for multiple testing (Table 1). As the number of samples was quite limited, Fisher’s exact test has been chosen for comparing SNP allelic frequencies between cases and unrelated controls, always using the genetic data from the 1000 Genomes Project (Phase 2) as reference population [42]. We also verified that the allelic frequencies for all the SNPs analyzed do not statistically deviate in our control data set from those reported in the 1000 Genomes Project EUR population. The moderate statistical association of rs3810688(T) with autism disappeared when comparing ASD data with those from Italian controls.

### 2.2. Haplotype Analysis (EUR Control Population)

A 6-SNPs haplotype block in *NLGN4X* showed a strong significant association with ASD cases (odds ratio = 3.58, *p*-value = 2.58 × 10^−6^), also after logistic regression and 100,000 permutation tests (empirical *p*-value < 1 × 10^−5^, empirical *q*-value < 1 × 10^−5^) (Table 2). This block (rs6638575(G), rs3810688(T), rs3810687(G), rs3810686(C), rs5916269(G), rs188260(T)) includes the SNP rs3810688(T), moderately associated with ASD in the single-locus analysis. Indeed, haplotypes on *NLGN4X* have been also analyzed as 5-, 4-, 3-, or 2-loci blocks, as reported in Table 2. A good statistical significance was obtained with blocks including the SNP rs3810688(T), especially together with the haplotype sets also including the SNPs rs3810687(G) and rs3810686(C)—both located in the 3′ UTR of *NLGN4X*—and with rs6638575(G), in the intron 5 of *NLGN4X* (Table 2)*.* These variants are part of two 5-loci (rs6638575(G), rs3810688(T), rs3810687(G), rs381086(C), rs5916269(G)) and (rs3810688(T), rs3810687(G), rs381086(C), rs5916269(G), rs1882260(T)), two 4-loci (rs6638575(G), rs3810688(T), rs3810687(G), rs381086(C)) and (rs3810688(T), rs3810687(G), rs3810686(C), rs5916269(G)), two 3-loci (rs6638575(G), rs3810688(T), rs3810687(G)) and (rs3810688(T), rs3810687(G), rs3810686(C)) and two 2-loci (rs6638575(G), rs3810688(T)) and (rs3810688(T), rs3810687(G)) combinations, all with strong statistical association with ASD. Moreover, despite the single-locus analysis not showing any statistical difference for the SNPs rs1882260 and rs5916269, haplotype analysis showed that two 2-blocks (rs5916269(G), rs1882260(T)) and (rs3810686(C), rs5916269(G)), two 3-blocks (rs3810686(C), rs5916269(G), rs1882260(T)) and (rs3810687(G), rs3810686(C), rs5916269(G)) and one 4-block loci (rs3810687(G), rs3810686(C), rs5916269(G), rs1882260(T)), respectively, have also a very strong significant association with ASD (Table 2). Similar results have been obtained also comparing the ASD haplotype frequencies of *NLGN4X* with our unrelated controls, suggesting the reliability of the selected control population for the statistical analysis (EUR Phase 2, 1000 Genomes Project).

No statistically significant haplotypes have been found in the *NLGN3* after logistic regression and permutation analysis.

### 2.3. Haplotype Analysis (ITA control Population)

The analysis was then repeated comparing the frequencies of *NLGN4X* SNPs between ASD cases and 1061 healthy controls collected during the Hypergenes project [43]. The association with the 6-marker haplotype remained, although weaker than the EUR population (odds ratio = 2.42, *p*-value = 6.33 × 10^−3^, empirical *p*-value = 6.79 × 10^−3^, empirical *q*-value < 2.73 × 10^−2^). A weaker association was also found for most of the 5-, 4-, 3- and 2-SNP haplotypes identified considering the EUR population. Data are reported in Table 3.

### 2.4. Linkage Disequilibrium Analysis

**Figure 1:**
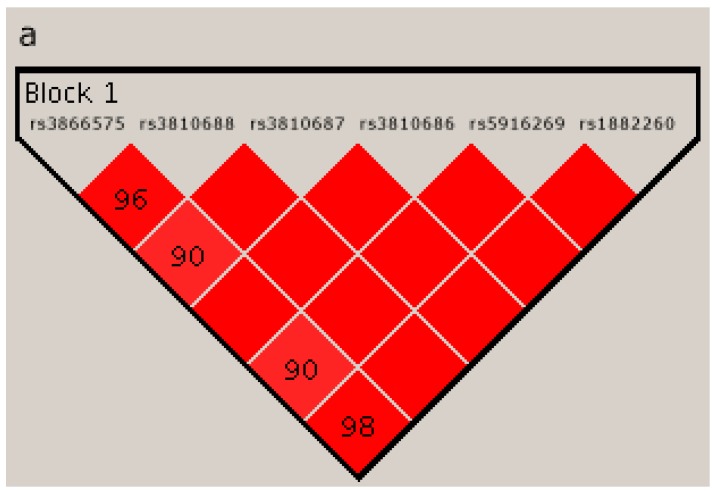

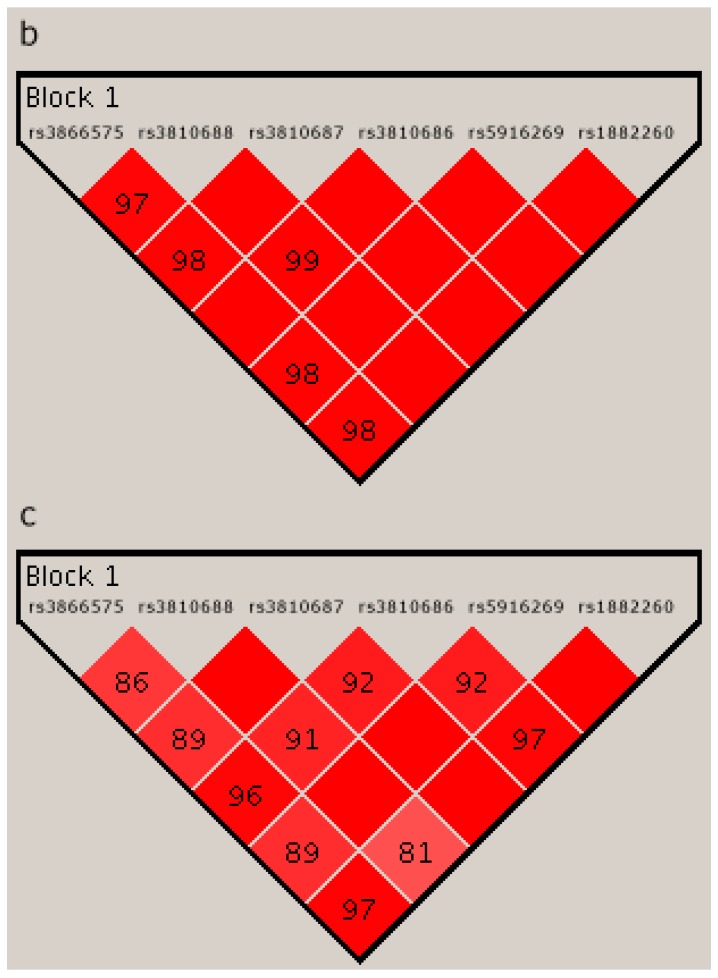
Linkage disequilibrium (LD) for the SNPs analyzed in *NLGN4X* calculated using Haploview: in panel (**a**) for the EUR population (1000 Genomes Project, Phase 2); in panel (**b**) for the Italian control population; and in panel (**c**) for the autistic patients enrolled in our study.

The results for linkage disequilibrium (LD) analysis for the considered SNPs in *NLGN4X* are presented in Figure 1. Panel (a) represents the LD for SNPs in the EUR control populations; panel (b) shows the LD scores for the ITA control population patients; panel (c) reports the results achieved for ASD patients.

## 3. Discussion

According to our results, common noncoding variants in *NLGN4X* may play a role in ASD susceptibility. Starting from a relatively homogenous sample and a case-control study design, our results suggest that some SNP marker haplotype blocks, located in the 3′ UTR of *NLGN4X*, might be associated to ASD. After a first statistical analysis, referring to EUR 1000 Genomes Project population as control, the 3′ UTR of *NLGN3* did not show any association with ASD, nor at single SNP or at haplotype blocks. On the other hand, the 3′ UTR of *NLGN4X* displayed statistical association with the disease at single SNP rs3810688(T), which is the minor allele (Table 1), although this significance was lost after FDR correction. Indeed, although the frequency of this allele is higher in the ASD population in comparison to EUR and ITA controls, the allele is still present in 21% of EUR population, thus producing only a modest effect by itself.

To confirm the statistical association of the haplotype blocks with ASD, (Table 2), we repeated the same statistical analyses, referring to the ITA healthy population, and confirmed the association, although weaker, between some SNP marker haplotypes in the 3′ UTR of *NLGN4X* and ASD. Indeed, the haplotype analysis revealed a 6-SNPs haplotype block (rs6638575(G), −rs3810688(T), −rs3810687(G), −rs3810686(C), −rs5916269(G), −rs1882260(T)) with a significant association with ASD, even after correction by permutation both referring to EUR (Table 2) and ITA control cohort populations (Table 3). In particular, in the region spanning from SNPs rs6638575 to rs3810686, several multiloci haplotype blocks were found to be associated with ASD (Table 2 and Table 3). Within the haplotype blocks containing rs3810687 and rs3810686, both SNPs always displayed the G allele. Interestingly, considering the LD analysis, we notice that the considered SNPs on *NLGN4X* are more in linkage than in control populations (Figure 1).

These results might imply that an approximately 4 kb region in the 3′ UTR of *NLGN4X*, spanning from SNPs rs66385775 to rs1882260, could be involved in autism susceptibility. This regulatory region in *NLGN4X* might have a role in modulating neuroligin biological function and, therefore, in causing or predisposing to neurological disorders. Since *NLGN4X* is involved in synaptic plasticity and X-linked, it might be a key gene in ASD pathogenesis and in its sex bias.

On the other hand, no associations were found for variants located on *NLGN3* at the single locus and haplotype analysis, despite Yu and coworkers (2011) reporting a positive association for the variants rs11795613(A), rs4844285(G), and rs4844286(T) with ASD [6]. Differences in sample ethnicity and size, as well as the ASD heterogeneity, might be taken into account for explaining such different results. Further studies, performed on a greater number of patients and with different genetic backgrounds, will be required to elucidate the role of *NLGN3* in autism susceptibility.

Nonetheless, our results are partially in agreement with Qi and collaborators (2009) that reported a positive association between SNPs in *NLGN4X* (rs6638575, rs3810686, rs1882260) and NSMR in a Chinese population [36]. In this regard, four putative haplotype combinations (rs3810686(T), rs1882260(T)), (rs3810686(C), −rs1882260(C)) and (rs6638575(G), rs3810686(T), rs18882260(T)) and (rs6638575(A), rs3810686(C), rs18882260(C)), in the sixth exon of *NLGN4X* have been reported as positively associated to NSMR in this Chinese cohort [36]. This genomic region, in fact, plays a crucial role for the biological function of *NLGN4X*, encoding for the transmembrane domain and postsynaptic density 95-disc large zone occludens-1 binding domain, both necessary for N-methyl aspartate receptors and downstream signal-transducing proteins. Furthermore, it has been reported that a target haplotype composed of the SNPs rs5916271(C) and rs6638575(A) had a significant effect in improving the general cognitive ability and especially verbal comprehension in male children, although further studies are required to highlight the role of these polymorphisms in ASD susceptibility [37].

As none of the analyzed SNPs on *NLGN4X* produce amino acid changes, two possible explanations should be taken into account for their association with ASD. First, considering the regulatory role of the 3′ UTR regions in modulating gene expression, the SNPs located in this region, correlated with ASD, might affect miRNA-binding sites, leading to impairments in *NLGN4X* expression. In this regard, the SNPs analyzed in this study might be located in or very close to putative miRNA regulatory regions, breaking or creating miRNA-binding sites with a possible effect on *NLGN4X* expression and thus on synaptic transmission. To this respect, it has been reported that a single nucleotide variant located in the 5′ UTR of *NLGN4X* is able to affect neuroligin expression [32], but further studies are required to validate if such a regulatory mechanism might also occur for the genetic variants in the 3′ UTR of neuroligin genes. Second, it might be also possible that other genetic variants, as putative predisposing loci or causative mutations, could exist in close linkage with the SNPs analyzed on *NLGN4X*, leading to differences in *NLGN4X* expression through alternative splicing or amino acid variations since each of these conditions can alter the biological function of *NLGN4X*.

Considering that males have higher predisposition in ASD etiology, an involvement of the X-chromosome in autism susceptibility has been suggested by genetic and cytogenetic studies [9,10,11,12,13] and several putative candidate genes, including neuroligins, have been largely investigated [7,8,23,24,25,26,27,28,29,30,31,32,33,34,35,36,37]. Nonetheless, taking advantages of the new sequencing technologies, further insights might be provided in discovering new X-linked genetic variants associated with ASD predisposition or its etiology.

Although the sample size of patients is quite limited for a genetic association study, statistical association between marker haplotype blocks located at 3′ UTR of *NLGN4X* and ASD was found referring both to EUR and ITA populations. Indeed, despite the stronger statistical significance found in reference to EUR controls, similar results were obtained in reference to ITA controls.

After all, considering the heterogeneity of ASD, in terms of symptoms, severity, and comorbidities, it sounds reasonable that several genes might be involved in ASD predisposition, as well as epigenetics or environmental factors. For this reason, a single genetic variant or haplotype, even if in a regulatory region or in a candidate gene, seems unlikely to be playing a major role in ASD predisposition. Thus, further functional and genetic studies coupled to these SNP profiles in a larger ASD cohort would be the next steps to elucidate the role of neuroligins and genetic variants in ASD etiology. Indeed, these SNPs could be the markers of other genetic variations or associated to specific phenotypes. Moreover, as gene–environment interaction has also been proposed for autism pathogenesis, further studies considering both the genetic and environmental risk factors should be designed.

As for clinical application, the relation between these genetic risk factors and symptoms or ASD characteristics could suggest molecular and epigenetic mechanisms triggering the disease and suggest possible preventive or therapeutic intervention.

In our study we found and discussed the association of several noncoding genetic variants in the *NLGN4X* gene with ASD in an Italian cohort. This implies that neuroligins and, therefore, impairments at the synaptic transmission, might play a role in ASD susceptibility and pathophysiology. To date, no other works have investigated the role of neuroligin noncoding variants in ASD susceptibility in an Italian autistic population, although Blasi and coworkers (2006) failed to find a significant association with ASD in neuroligin coding variants [39] when analyzing Italian ASD patients. Taking into account the contradictory results in validating the role of neuroligins in ASD predisposition as well as the complex etiology of this disease, our findings can be important for the characterization of this disease, although they need to be confirmed and replicated in different ethnic groups and to be associated with phenotypic and epigenetic data.

## 4. Materials and Methods

### 4.1. Subjects

The sample-sets consist of: 202 ASD patients (165 (81.7%) males; 37 (18.3%) females), recruited by IRCCS Eugenio Medea-La Nostra Famiglia in two different and far areas of Italy: Bosisio Parini (Lecco, Italy) in the North and Ostuni (BR, Italy) in the South. Patients had an age ranging between 2 and 12 years. Demographic characteristics are summarized in Table 4.

The diagnosis for young autism (70.3%), Asperger’s syndrome (3.5%), or PDD-NOS (26.2%) were performed according to DSM-IV TR (APA, 2000) and by the Autism Diagnostic Observation Scale (ADOS) and ADI-R. IQ, cognitive tests, and behavioral analyses were also done in all the patients, but 20, and are summarized in Table 5. Patients with genetic syndromes, epilepsy, and neuroradiological confirmed disorders were excluded.

One thousand sixty one healthy controls, collected during the Hypergenes Project [43] (European Network for Genetic-Epidemiological Studies; www.hypergenes.eu) were included in the study. These controls were from the same two areas of Italy from which patients were recruited and in similar proportion. The healthy subjects were genotyped at the University of Milan, using the Illumina 1M-duo array (San Diego, CA, USA) and imputed with Minimac software [44], a low-memory, fast, flexible, computationally efficient implementation of the Markov chain haplotyping method, using the haplotypes from the 1000 Genomes Project (March 2012) as reference [42].

The data relative to the SNPs considered in the statistical analyses were obtained directly from genotyping for rs5916269, rs3810686, rs3810687, and rs663857 and by imputation for rs1882260 and rs3810688.

The Ethical committees of IRCCS Eugenio Medea-La Nostra Famiglia and of Hypergenes project approved the study.

Moreover, genotype data from the 1000 Genomes Project (Phase 2) [42] were also used as control cases, considering the 379 individuals with EUR genotype.

### 4.2. Selection of SNPs

The NCBI Accession Numbers, used as reference sequences for *NLGN3* and *NLGN4X* were, respectively, NG_015874 and NG_008881. Relying on literature [6,36], a selection of SNPs located in the intronic or 3′ UTR regions of both *NLGN3* and *NLGN4X* were genotyped. Furthermore, three additional SNPs, located in the 3′ UTR of *NLGN4X*, have been included since they are located in a putative regulatory region and have a close map distance with the previously selected SNPs. In this regard, the inter-SNP distance, for the SNPs analyzed in *NLGN3* and *NLGN4X*, was less than 5 kb. All the studied SNPs have a minor allele frequency (MAF) >0.05—as reported by the 1000 Genomes Project (Phase 2) in the EUR population—and are listed in the Table 6. Linkage disequilibrium for SNPs considered in this study has been calculated using Haploview [45], and the most relevant LD scores for these data are displayed in Figure 1. Figure S1 represents the LD scores (derived from the 1000 Genomes Project EUR population) of all the SNPs located in *NLGN4X*, including those selected for our analyses and the related TAG SNPs.

### 4.3. SNPs Screening and Genotyping

Genomic DNA was extracted from peripheral blood samples, using commercial kit (Macherey-Nagel GmbH & Co. KG, Düren, Germany). SNPs located on both *NLGN3* and *NLGN4X* were amplified and genotyped by high-resolution melting analysis (HRM) (Rotor-Gene^®^ Q-Pure detection, Qiagen, Venlo, The Netherlands). For each sample a total of 20 ng of genomic DNA was amplified in a 20 μL reaction mixture Phusion Flash High Fidelity PCR Master Mix (Thermo Fisher Scientific, Waltham, MA, USA), Stati Uniti of primer mix (0.7 each) and 5% of the intercalating dye EvaGreen (Biotium Inc., Fremont, CA, USA), with 1.4, as final concentration. HRM analysis primers were designed by PRIMER3plus software (http://bioinfo.ut.ee/primer3-0.4.0/primer3/). All samples were analyzed in duplicates, and for each selected SNP three internal controls—resembling all three possible genotypes, previously characterized by Sanger-sequencing—have been included. The temperature raising for the HRM analysis was of 0.1 °C/s. Normalized melting curves and differential graphs have been compared for the genotype analysis. Primers and amplification details are shown in the Table S5.

### 4.4. Statistical Analysis

A first screening was performed comparing the data obtained from ASD patients with those from the European subset of the 1000 Genomes Project (EUR Phase 2 population). SNP association analyses were performed first using Plink v1.07 [46] to test for possible associations between all the SNPs identified in *NLGN3* and *NLGN4X* and ASD. The two-tailed Fisher’s exact test was chosen to compare the polymorphisms’ distributions (cases vs. controls) and testing their significance at *p* < 0.05, under the null hypothesis of no association between each SNP and ASD. The *p*-values have been adjusted by false discovery rate (FDR) correction for multiple test analysis.

Using the same control data, haplotype tests were also performed using Plink v1.07. Omnibus multiple loci analyses were carried out for all the SNPs included in this study regarding *NLGN3* and *NLGN4X*, using logistic regression under the null hypothesis of no association between haplotypes and ASD. Permutation tests (100,000 permutations) were used to correct the *p*-values. Haplotypes with *p*-values equal or below the α threshold of 0.05 were considered as statistically significant.

The same statistical analyses were performed on SNPs in *NLGN4X*, which revealed a significant correlation with ASD, considering two groups of Italian healthy controls recruited in the same two areas of Italy in which patients were recruited.

## Figures and Tables

**Table 1:** Single locus analysis for all the single nucleotide polymorphisms (SNPs) genotyped in *NLGN3* and *NLGN4X* (EUR control population).

Gene ID	SNP ID	Minor Allele	MAF Controls	MAF Case	*p*-Value	OR	95% CI	*p*-Corr
*NLGN3*	rs11795613	G	0.481	0.515	0.398	1.144	0.816–1.617	0.701
	rs4844285	A	0.4741	0.4895	0.701	1.064	0.755–1.496	0.701
	rs4844286	T	0.505	0.481	0.5396	0.908	0.646–1.280	0.701
*NLGN4X*	rs6638575	A	0.266	0.301	0.303	1.193	0.856–1.663	0.404
	rs3810688	T	0.2176	0.288	0.0456	1.453	1.018–2.074	0.152
	rs3810687	T	0.1086	0.1339	0.3369	1.269	0.805–2.000	0.404
	rs3810686	T	0.4466	0.4561	0.817	1.039	0.768–1.407	0.817
	rs5916269	A	0.1086	0.1339	0.3369	1.269	0.805–2.000	0.404
	rs1882260	C	0.2655	0.3347	0.0505	1.392	1.005–1.928	0.152

Statistical significance, for the single locus association analysis, was tested at *p* < 0.05, after Fisher’s exact test. The *p*-values have been adjusted by false discovery rate (FDR) correction for multiple test analysis. MAF controls, frequency of the minor allele in controls (*n* = 379); MAF case, frequency of the minor allele in case (*n* = 202); OR, odds ratio; CI, confidence interval; *p*-corr, corrected *p*-value after FDR test.

**Table 2:** Haplotype association analysis in *NLGN4X* (*EUR control population*).

N	SNPs in Haplotypes	Control Frequency	Case Frequency	Haplotype	*p*-Value	Emp. *p*-Value	Emp. *q*-Value
6	rs6638575-rs3810688-rs3810687-rs3810686-rs5916269-rs1882260	0.0335	0.1799	GTGCGT	2.58 × 10^−6^	<1 × 10^−5^	<1 × 10^−5^
5	rs6638575-rs3810688-rs3810687-rs3810686-rs5916269	0.0805	0.1799	GTGCG	7.60 × 10^−6^	<1 × 10^−5^	<1 × 10^−5^
5	rs3810688-rs3810687-rs3810686-rs5916269-rs1882260	0.0335	0.1799	TGCGT	2.58 × 10^−6^	<1 × 10^−5^	<1 × 10^−5^
4	rs6638575-rs3810688-rs3810687-rs3810686	0.0805	0.1799	GTGC	7.6 × 10^−6^	<1 × 10^−5^	2 × 10^−5^
4	rs3810688-rs3810687-rs3810686-rs5916269	0.0801	0.1793	TGCG	7.6 × 10^−6^	<1 × 10^−5^	2 × 10^−5^
4	rs3810687-rs3810686-rs5916269-rs1882260	0.0943	0.1793	GCGT	1.3 × 10^−5^	<1 × 10^−5^	2 × 10^−5^
3	rs6638575-rs3810688-rs3810687	0.3538	0.4463	GTG	8.16 × 10^−6^	<1 × 10^−5^	<1 × 10^−5^
3	rs3810688-rs3810687-rs3810686	0.0701	0.1793	TGC	7.6 × 10^−6^	<1 × 10^−5^	<1 × 10^−5^
3	rs3810687-rs3810686-rs5916269	0.4062	0.4445	GCG	8.16 × 10^−4^	6.99 × 10^−4^	3.2 × 10^−3^
3	rs3810686-rs5916269-rs1882260	0.0943	0.1793	CGT	1.3 × 10^−5^	<1 × 10^−5^	3 × 10^−4^
2	rs6638575-rs3810688	0.2109	0.2854	GT	1.08 × 10^−4^	7 × 10^−5^	8.3 × 10^−4^
2	rs3810688-rs3810687	0.0943	0.1793	TG	1.3 × 10^−5^	2 × 10^−5^	1.5 × 10^−4^
2	rs3810687-rs3810686	0.4062	0.4445	GC	8.16 × 10^−4^	7.3 × 10^−4^	5 × 10^−3^
2	rs3810686-rs5916269	0.4062	0.4445	CG	8.16 × 10^−4^	7.3 × 10^−4^	5 × 10^−3^
2	rs5916269-rs1882260	0.4314	0.6259	GT	2.4 × 10^−8^	<1 × 10^−5^	<1 × 10^−5^

Haplotype combinations with significant *p*-values (*p* < 0.05)*,* using the EUR control population, after permutation test, have been reported for all the SNPs located on *NLGN4X.* Empirical *p*-values and empirical *q*-values after 100,000 permutations are reported. Only haplotypes having case frequency >0.05 have been presented.

**Table 3:** Haplotype association analysis in *NLGN4X* (ITA control population).

N	SNPs in Haplotypes	Control Frequency	Case Frequency	Haplotype	*p*-Value	Empirical *p*-Value	Empirical *q*-Value
6	rs6638575-rs3810688-rs3810687-rs3810686-rs5916269-rs1882260	0.0678	0.1799	GTGCGT	6.33 × 10^−3^	6.79 × 10^−3^	2.73 × 10^−3^
5	rs6638575-rs3810688-rs3810687-rs3810686-rs5916269	0.1349	0.1799	GTGCG	1.9 × 10^−2^	1.87 × 10^−2^	4.47 × 10^−2^
5	rs3810688-rs3810687-rs3810686-rs5916269-rs1882260	0.0678	0.1799	TGCGT	6.33 × 10^−3^	5.39 × 10^−3^	2.78 × 10^−2^
4	rs6638575-rs3810688-rs3810687-rs3810686	0.1189	0.1799	GTGC	1.66 × 10^−2^	1.62 × 10^−2^	4.41 × 10^−2^
3	rs6638575-rs3810688-rs3810687	0.3782	0.4463	GTG	1.56 × 10^−2^	1.29 × 10^−2^	4.37 × 10^−2^
3	rs3810688-rs3810687-rs3810686	0.0801	0.1793	TGC	1.17 × 10^−2^	1.26 × 10^−2^	4.78 × 10^−2^
3	rs3810687-rs3810686-rs5916269	0.3360	0.4445	GCG	3.99 × 10^−3^	4.10 × 10^−3^	1.98 × 10^−2^
2	rs3810687-rs3810686	0.3160	0.4445	GC	3.50 × 10^−3^	3.20 × 10^−3^	2.20 × 10^−2^
2	rs3810686-rs5916269	0.3360	0.4445	CG	3.99 × 10^−3^	3.70 × 10^−3^	2.53 × 10^−2^

Haplotype combinations with significant *p*-values (*p* < 0.05), using the ITA control population, after permutation test, have been reported for all the SNPs located on *NLGN4X*. Empirical *p*-values and empirical *q*-values after 100,000 permutations are reported. Only haplotypes having case frequency >0.05 have been presented.

**Table 4:** Demographic data and diagnosis of autistic patients.

Patients: 202; Age: 2–12; Males: 165; Females: 37		
Patients from North Italy: *n* = 157	Males: 131, 83.44%	Young autism: 93 (71%)
		PDD-NOS: 34 (26%)
		Asperger’s Syndrome: 4 (3%)
	Females: 26, 16.56%	Young autism: 18 (69.2%)
		PDD-NOS: 7 (27%)
		Asperger’s Syndrome: 1 (3.8%)
Patients from South Italy: *n* = 45	Males: 34, 75.55%	Young autism: 24 (70.6%)
		PDD-NOS: 9 (26.5%)
		Asperger’s Syndrome: 1 (2.9%)
	Females: 11, 24.45%	Young autism: 8 (72.7%)
		PDD-NOS: 3 (27.3%)
		Asperger’s Syndrome: 0

**Table 5:** Cognitive and behavioral characteristics of autistic patients.

IQ		Hyperactivity	Language and Communication
>70 *n* = 71 (35.1%)	Level 1: IQ > 101; *n* = 15 (7.4%)	Level 0: *n* = 136; (67.3%)	Level 1: *n* = 7 (3.5%)
	Level 2: 100 > IQ> 70; *n* = 56 (27.7%)		Level 2: *n* = 48 (23.7%)
≤69 *n* = 131 (64.6%)	Level 3: 69 > IQ > 50; *n* = 79 (39.1%)	Level 1: *n* = 53 (26.2%)	Level 3: *n* = 65 (32.2%)
	Level 4: 49 > IQ > 35; *n* = 28 (13.9%)		Level 4: *n* = 64 (31.7%)
	Level 5: 34 > IQ> 20; *n* = 17 (8.4%)	Level 2: *n* = 13 (6.5%)	Level 5: *n* = 3 (1.5%)
	Level 6: IQ < 19; *n* = 7 (3.5%)		15 patients not evaluated (7.4%)

**Table 6:** List of SNPs analyzed in *NLGN3* and *NLGN4X*.

Genes	SNPs	Alleles	MAF	Position in Gene	References
*NLGN3*	rs11795613	(A/G)	G: 0.49	Intron 1	Yu et al., *Behav. Brain Funct.* 2011, *7*, 13 [6]
	rs4844285	(A/G)	A: 0.48	Intron 2	
	rs4844286	(T/G)	T: 0.49	Intron 2	
*NLGN4X*	rs6638575	(A/G)	A: 0.28	Intron 5	Qi et al., *Psychiatr. Genet.* 2009, *19*, 1 [36]
	rs3810686	(T/C)	T: 0.44	3′ UTR	
	rs1882260	(C/T)	C: 0.27	3′ UTR	
	rs3810687	(T/G)	T: 0.11	3′ UTR	–
	rs3810688	(T/C)	T: 0.29	3′ UTR	
	rs5916269	(A/G)	A: 0.11	3′ UTR	

All the SNPs analyzed as well as their position along the *NLGN3* and *NLGN4X* genes and MAF, are listed. MAF is referred to that reported for the EUR population (1000 Genomes Project, Phase 2). According to literature, their association to autism or psychiatric conditions, has been also reported. SNP: Single Nucleotide Polymorphism; MAF: Minor Allele Frequency; UTR: Untranslated Region.

## Supplementary Materials

Supplementary materials can be found at www.mdpi.com/1422-0067/17/10/1765/s1.

## Abbreviations

ASD	Autism spectrum disorder
EUR	European
FDR	False discovery rate
HRM	High resolution melting
IQ	Intelligence Quotient
ITA	Italian
MAF	Minor allele frequency
NLGN	Neuroligin
NSMR	Non-syndromic mental retardation
PDD-NOS	Pervasive developmental disorder-not otherwise specified
SNP	Single nucleotide polymorphism
