# Supplementary material for: Association Analysis of Noncoding Variants in Neuroligins 3 and 4X Genes with Autism Spectrum Disorder in an Italian Cohort
Source: Int J Mol Sci. 2016 Oct 22;17(10):1765. doi: 10.3390/ijms17101765 (PMC5085789; doi:10.3390/ijms17101765)
Supplement: Supplementary file 1 [file ijms-17-01765-s001.pdf]

Supplementary Materials: Association Analysis of Non Coding Variants in Neuroligins 3 and 4X Genes with Autism Spectrum Disorder in an Italian Cohort

Martina Landini, Ivan Merelli, M. Elisabetta Raggi, Nadia Galluccio, Francesca Ciceri, Arianna Bonfanti, Serena Camposeo, Angelo Massagli, Laura Villa, Erika Salvi, Daniele Cusi, Massimo Molteni, Luciano Milanesi, Anna Marabotti and Alessandra Mezzelani

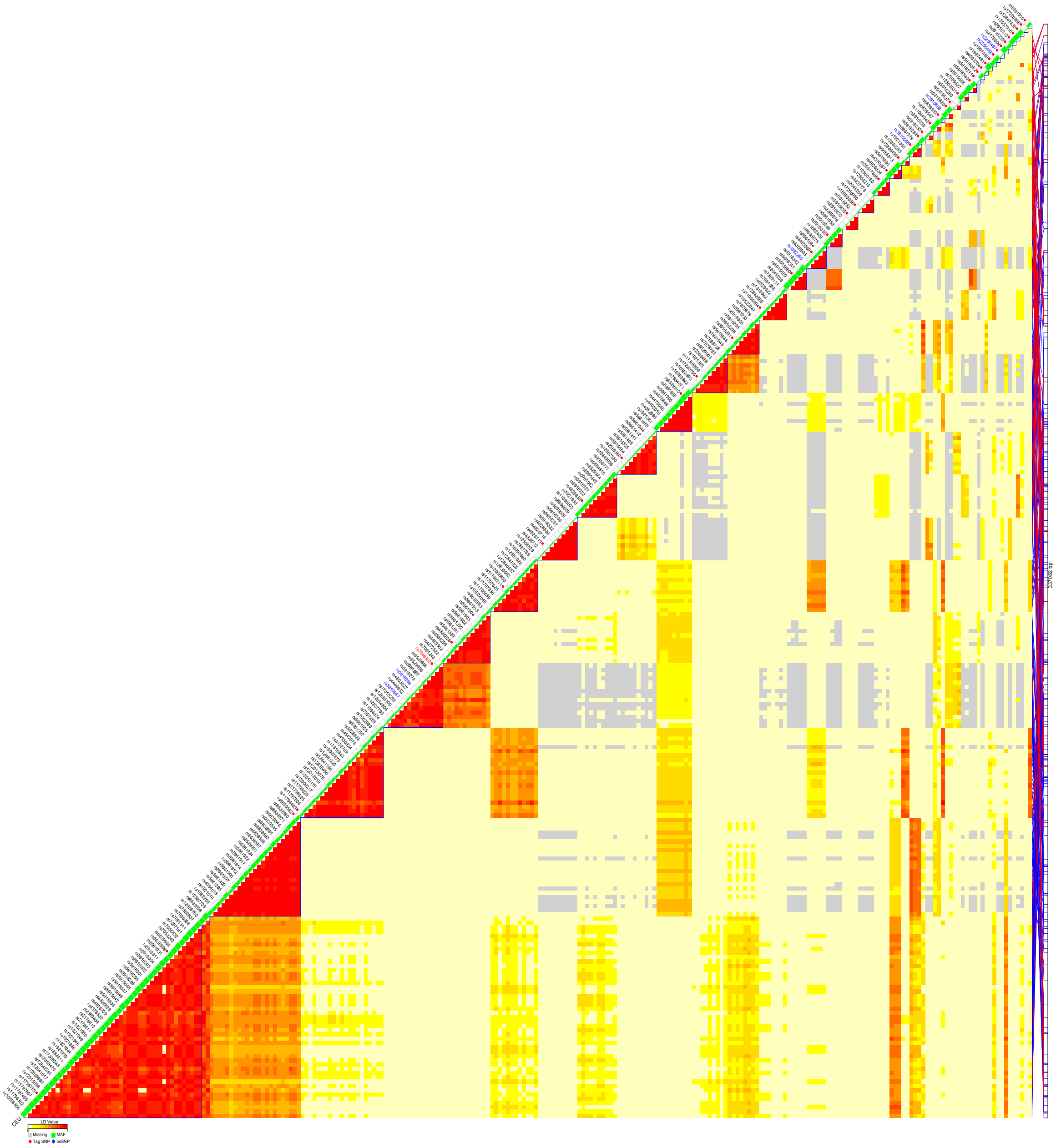

Figure S1. Represents the LD scores, derived from the 1000 Genome Project EUR population, of all the SNPs located in NLGN4X including those selected for our analyses and the related TAG SNPs.

Table S1. Hardy-Weinberg equilibrium considering the EUR control population.

SNP	Test	A1	A2	GENO	Observed Heterozygosity	Expected Heterozygosity	*p*-Value
rs6638575	ALL	A	G	22/93/123	0.3908	0.41	0.5264
rs6638575	AFF	A	G	7/13/17	0.3514	0.4635	0.1598
rs6638575	UNAFF	A	G	15/80/106	0.398	0.3975	1
rs3810688	ALL	T	C	21/84/133	0.3529	0.3893	0.1812
rs3810688	AFF	T	C	03/07/27	0.1892	0.2896	0.05608
rs3810688	UNAFF	T	C	18/77/106	0.3831	0.4042	0.4852
rs3810687	ALL	T	G	6/41/191	0.1723	0.1979	0.05161
rs3810687	AFF	T	G	02/07/28	0.1892	0.2531	0.1544
rs3810687	UNAFF	T	G	4/34/163	0.1692	0.1871	0.2417
rs3810686	ALL	T	C	56/103/79	0.4328	0.4953	0.05063
rs3810686	AFF	T	C	10/14/13	0.3784	0.4967	0.185
rs3810686	UNAFF	T	C	46/89/66	0.4428	0.495	0.1537
rs5916269	ALL	A	G	6/41/191	0.1723	0.1979	0.05161
rs5916269	AFF	A	G	02/07/28	0.1892	0.2531	0.1544
rs5916269	UNAFF	A	G	4/34/163	0.1692	0.1871	0.2417
rs1882260	ALL	C	T	22/92/124	0.3866	0.4082	0.4278
rs1882260	AFF	C	T	08/12/17	0.3243	0.4704	0.07819
rs1882260	UNAFF	C	T	14/80/107	0.398	0.393	1

Genotype counts and Hardy-Weinberg test statistics for each SNP considering the EUR control population. ALL = all subjects; AFF = affected; UNAFF = unaffected.

Table S2. Hardy-Weinberg equilibrium considering the ITA control population.

SNP	Test	A1	A2	GENO	Observed Heterozygosity	Expected Heterozygosity	*p*-Value
rs6638575	ALL	A	G	62/188/222	0.3983	0.4425	0.0294
rs6638575	AFF	A	G	7/13/17	0.3514	0.4635	0.1598
rs6638575	UNAFF	A	G	55/175/205	0.4023	0.4405	0.08112
rs3810688	ALL	T	C	25/178/269	0.3771	0.3664	0.6153
rs3810688	AFF	T	C	03/07/27	0.1892	0.2896	0.05608
rs3810688	UNAFF	T	C	22/171/242	0.3931	0.3721	0.3019
rs3810687	ALL	T	G	10/100/362	0.2119	0.2219	0.3019
rs3810687	AFF	T	G	02/07/28	0.1892	0.2531	0.1544
rs3810687	UNAFF	T	G	8/93/334	0.2138	0.2192	0.6596
rs3810686	ALL	T	C	84/232/156	0.4915	0.4884	0.9251
rs3810686	AFF	T	C	10/14/13	0.3784	0.4967	0.185
rs3810686	UNAFF	T	C	74/218/143	0.5011	0.4874	0.6228
rs5916269	ALL	A	G	11/100/361	0.2119	0.2251	0.2168
rs5916269	AFF	A	G	02/07/28	0.1892	0.2531	0.1544
rs5916269	UNAFF	A	G	9/93/333	0.2138	0.2226	0.3893
rs1882260	ALL	C	T	64/189/219	0.4004	0.4461	0.05996
rs1882260	AFF	C	T	08/12/17	0.3243	0.4704	0.07819
rs1882260	UNAFF	C	T	56/177/202	0.4069	0.4437	0.08437

Genotype counts and Hardy-Weinberg test statistics for each SNP considering the ITA control population. ALL = all subjects; AFF = affected; UNAFF = unaffected.

Table S3. Single locus analysis for male individuals considering all the SNPs genotyped in *NLGN3* and *NLGN4X* (EUR control population).

Gene	SNP	BP	A1	F_A	F_U	P	OR
NLGN3	rs11795613	8,684,936	G	0.5152	0.4775	0.1862	1.163
NLGN3	rs4844285	8,687,852	A	0.497	0.4663	0.5699	1.131
NLGN3	rs4844286	8,689,182	T	0.5212	0.4775	0.1188	1.191
NLGN4X	rs6638575	339,066	A	0.2727	0.2472	0.5898	1.142
NLGN4X	rs3810688	340,951	T	0.3034	0.2364	0.0431	1.406
NLGN4X	rs3810687	341,023	T	0.1273	0.118	0.793	1.09
NLGN4X	rs3810686	341,133	T	0.4545	0.4382	0.7609	1.068
NLGN4X	rs5916269	341,408	A	0.1273	0.118	0.793	1.09
NLGN4X	rs1882260	342,846	C	0.3152	0.2584	0.2453	1.321

Single locus analysis for male individuals considering all the SNPs genotyped in *NLGN3* and *NLGN4X*.

Table S4. Single locus analysis for female individuals considering all the SNPs genotyped in *NLGN3* and *NLGN4X* (EUR control population).

Gene	SNP	BP	A1	F_A	F_U	P	OR
NLGN3	rs11795613	8,684,936	G	0.5135	0.4826	0.6248	1.132
NLGN3	rs4844285	8,687,852	A	0.473	0.4776	0.9415	0.9816
NLGN3	rs4844286	8,689,182	T	0.4865	0.4975	0.8616	0.9568
NLGN4X	rs6638575	339,066	A	0.3649	0.2736	0.1112	1.525
NLGN4X	rs3810688	340,951	T	0.2811	0.1757	0.05889	1.834
NLGN4X	rs3810687	341,023	T	0.1486	0.1045	0.267	1.497
NLGN4X	rs3810686	341,133	T	0.4595	0.4502	0.8837	1.038
NLGN4X	rs5916269	341,408	A	0.1486	0.1045	0.267	1.497
NLGN4X	rs1882260	342,846	C	0.3784	0.2687	0.05485	1.657

Single locus analysis for female individuals considering all the SNPs genotyped in *NLGN3* and *NLGN4X*.

Table S5. SNP genotyping.

Gene	SNPs	Alleles	Primers	Amplification	HRMA (°C)
NLGN3	rs4844285	(A/G)	Forward: 5′TCTGAGGTTGGTAGGGTACAGT3′<br>Reverse: 5′CCTGCAGGTTTAAGAGACCTT3′	Hold: 98 °C × 15″; Cycling (×35):<br>98 °C × 3″/66 °C × 5″/72 × 7″<br>Touchdown: 0.5 °C × 20 cycles	74–85
	rs11795613	(A/G)	Forward: 5′GGCAGTGGATCATCTGTGGGA3′<br>Reverse: 5′AAGATGGTCTGGGAGGGCAA3′	Hold: 98 °C × 15″; Cycling (×40):<br>98 °C × 2″/70 °C × 5″/72 °C × 8″	79–90
	rs4844286	(T/G)	Forward: 5′AGCAACCCAGGAACACTTTCA3′<br>Reverse: 5′TTCCCCAGGAAACCCTTCAT3′	Hold: 98 °C × 10″; Cycling (×40):<br>98 °C × 2″/60 °C × 7″/72 °C × 8″	75–86
NLGN4X	rs6638575	(A/G)	Forward: 5′TGATTCTCCTTCTACCTGTGACC3′<br>Reverse: 5′AGACACCCAGAAACAAGTCA3′	Hold: 98 °C × 10″; Cycling (×40):<br>98 °C × 5″/59 °C × 20″/72 °C × 15″	70–90
	rs3810686	(T/C)	Forward: 5′GCAGACCCTTATCGTTGGTG3′<br>Reverse: 5′GCCTTGAAATGGTGATGTCCTA3′	Hold: 98 °C × 10″; Cycling (×40):<br>98 °C × 5″/58 °C × 20″/72 °C × 15″	70–90
	rs1882260	(C/T)	Forward: 5′CAACCTTTGATTACTGAATCCCTAC3′<br>Reverse: 5′CCCCAAATCCTATTGCTTATCTTG3′	Hold: 98 °C × 10″; Cycling (×40):<br>98 °C × 5″/59 °C × 20″/72 °C × 15″	70–90
	rs3810688	(T/C)	Forward: 5′CTATCCCTCTGCCCTACCCG3′<br>Reverse: 5′CCAACGATAAGGGTCTGCCG3′	Hold: 98 °C × 10″; Cycling (×40):<br>98 °C × 5″/58 °C × 20″/72 °C × 15″	75–86
	rs5916269	(A/G)	Forward:5′TTAAAAGAATGGACTGTGCAGCG3′<br>Reverse: 5′TAGTTCAGGCTGGCAAAACAC3′	Hold: 98 °C × 10″; Cycling (×40):<br>98 °C × 5″/58 °C × 20″/72 °C × 15″	70–90
	rs3810687	(T/G)	Forward: 5′GCAAAAAGGCAGTCATCCCA3′<br>Reverse: 5′TTCTCACATTTCACAGGGTCAGA3′	Hold: 98 °C × 10″; Cycling (×40):<br>98 °C × 5″/58 °C × 20″/72 °C × 15″	70–85

Primers and HRMA protocols for all the SNPs analyzed in this study. HRMA (°C): melting temperature range for High Resolution Melting Analysis.
